# Association between Internet addiction and depression in Thai medical students at Faculty of Medicine, Ramathibodi Hospital

**DOI:** 10.1371/journal.pone.0174209

**Published:** 2017-03-20

**Authors:** Thummaporn Boonvisudhi, Sanchai Kuladee

**Affiliations:** Department of Psychiatry, Faculty of Medicine, Ramathibodi Hospital, Mahidol University, Bangkok, Thailand; Ariel University, ISRAEL

## Abstract

**Objective:**

To study the extent of Internet addiction (IA) and its association with depression in Thai medical students.

**Methods:**

A cross-sectional study was conducted at Faculty of Medicine, Ramathibodi Hospital. Participants were first- to fifth-year medical students who agreed to participate in this study. Demographic characteristics and stress-related factors were derived from self-rated questionnaires. Depression was assessed using the Thai version of Patient Health Questionnaire (PHQ-9). A total score of five or greater derived from the Thai version of Young Diagnostic Questionnaire for Internet Addiction was classified as “possible IA”. Then chi-square test and logistic regression were used to evaluate the associations between possible IA, depression and associated factors.

**Results:**

From 705 participants, 24.4% had possible IA and 28.8% had depression. There was statistically significant association between possible IA and depression (odds ratio (OR) 1.92, 95% confidence interval (CI): 1.34–2.77, P-value <0.001). Logistic regression analysis illustrated that the odds of depression in possible IA group was 1.58 times of the group of normal Internet use (95% CI: 1.04–2.38, P-value = 0.031). Academic problems were found to be a significant predictor of both possible IA and depression.

**Conclusion:**

IA was likely to be a common psychiatric problem among Thai medical students. The research has also shown that possible IA was associated with depression and academic problems. We suggest that surveillance of IA should be considered in medical schools.

## Introduction

Internet addiction (IA), described as a loss of control over Internet use that impacts on daily life functions, relationships and emotional stability, is an emerging issue with growing interest nowadays [1]. Its characteristics comprises of four features: 1) excessive use, often related with a loss of sense of time; 2) withdrawal, including feelings of anger, tension, and/or depression if the network is inaccessible; 3) tolerance, including the need for better computer equipment or more hours of use; and 4) negative repercussions, such as poor achievement and social isolation [2,3]. It was found that more than 50% of individuals with IA reported themselves enduring severe impairment in academic, relationship, financial and occupational problems [4]. Across Asia and Europe, the prevalence of IA varies from 1% to 36.7% depending on different assessment tools and study populations [1]. However, the overall rate of IA is greater among college students as compared to general population and adolescents [5].

Previously, an association between IA and depression has been reported among adults [6], adolescents [7–9] and college students [10]. A systematic review of comorbid psychopathology in pathological Internet use similarly revealed that 75% of the examined studies reported a significant correlation between pathological Internet use and depression [11]. When compared to other psychiatric comorbidities, depression also showed the strongest correlation with pathological Internet use [11]. Consistently, a meta-analysis of five studies noted that the prevalence of depression among IA group was significantly higher than that of the control group [12].

As an effective way to access information and boundless communication with others, the Internet use has become commonplace especially among the young generation, including medical students. Nevertheless, losing control of its use can result in negative consequences, such as difficulty completing assignments, sleep deprivation, and poor academic performance [4,13]. Indeed, recent surveys from various countries have reported the prevalence of IA in medical students to be ranging from 5.5–44.6% [13–16]. The affected group reported more emotional symptoms [14] and also appeared to have poorer academic performance as well as lower quality of life in the domain of physical, psychological, and social relation compared to non-affected group. [17].

Therefore, IA becomes an important issue in medical training especially in the countries with inadequate number of medical practitioners such as Thailand. To solve this problem, the government has encouraged medical schools to train more number of students with highly expected performance after graduation. Nevertheless, medical students who suffer from IA or other psychiatric problems might not be able to achieve such qualification or take much more time in their study course or even drop-out. In such cases, a dramatic waste of time and money would be an inevitable cost for both the society and the students as well as their family.

To prepare for this emerging, high-impact problem, the extent of IA and its related conditions among Thai medical students should be elucidated. To date, no such data is available in Thailand. Since there is still no consensus on the clinical definition of IA [18], in this study we defined cases with possible IA according to a criterion originally proposed by Young [4] with the aims to illustrate the prevalence of possible IA and its association with depression among Thai medical students at the Faculty of Medicine, Ramathibodi Hospital.

## Methods

### Participants and procedure

This project is a cross-sectional descriptive study conducted at the Faculty of Medicine, Ramathibodi Hospital, Mahidol University, Bangkok, Thailand. All first- to fifth-year medical students studying at the Faculty of Medicine, Ramathibodi Hospital, during the academic year 2015, were recruited. Those who refused to participate in the study or to provide an informed consent were excluded. This study was approved by the Ethics Committee on Human Experimentation of the Faculty of Medicine, Ramathibodi Hospital (ID 05-58-54).

After giving a written informed consent, each participant completed a set of questionnaires comprising four parts: 1) demographic data; 2) factors associated with stress in medical students as proposed by Saipanish [19], which include academic problems, difficulty in peer and love relationships, health problems, financial problems, family problems, and conflict with teachers; 3) the Thai version of Young Diagnostic Questionnaire for Internet Addiction; and 4) the Thai version of Patient Health Questionnaire (PHQ-9).

### Measures

#### Young Diagnostic Questionnaire for Internet Addiction

Young Diagnostic Questionnaire for Internet Addiction is a self-reported instrument developed for screening of IA [4]. It consists of eight items asking about Internet use. The questions were adapted from the *Diagnostic and Statistical Manual of Mental Disorders*, 4th edition (DSM-IV) criteria for pathological gambling. The definition of “Internet use” in the questionnaire includes all activities in using the Internet. Each item is scored as 0 (“no”) or 1 (“yes”), and the total score ranges from 0 to 8. In the original study conducted by Young [4], a total score of five or above was considered to be dependent Internet user, similar to the cutoff criterion used to diagnose pathological gambling. At this cut-off point, the Thai version of Young Diagnostic Questionnaire for Internet Addiction demonstrated acceptable internal consistency (Cronbach’s alpha = 0.71) [20]. In this study, the participants whose total score ranging from 5 to 8 were classified as “possible IA”.

#### Patient Health Questionnaire (PHQ-9)

The Patient Health Questionnaire (PHQ-9) [21] has been widely used as a self-administered screening tool for depression. All nine items are designed to parallel the symptom in diagnostic criteria for major depressive episode according to the DSM-IV. The scoring for each component is based on the symptom experienced during the past two weeks before the evaluation and is defined as follows: 0 for “not at all”, 1 for “several days”, 2 for “more than half of the days”, and 3 for “nearly every day”. The total score ranges from 0 to 27. The Thai version of the PHQ-9, at a cut-off score of 9 or greater, exhibited good performance with a sensitivity of 0.84 and a specificity of 0.77. The tool also has satisfactory internal consistency (Cronbach’s alpha = 0.79) [22].

### Statistical analyses

Data analyses were conducted by IBM SPSS Statistics for Windows, Version 22.0 (IBM Corp., Armonk, NY, USA). Characteristics of the participants were reported as percentages. Chi-square test was applied for analyses of associations among possible IA, depression, demographic data, and factors associated with stress in medical students. The associations between each item of the Young Diagnostic Questionnaire for Internet Addiction and depression were also analyzed using Chi-square statistic. Finally, logistic regression was used to determine predictors of possible IA and depression.

## Results

Of all 905 medical students, 705 (78%) students participated in this study. Demographic characteristics and their associations with possible IA and depression are shown in Table 1. The mean age of the participants was 20.51 years old (standard deviation (SD) = 1.91). The mean Young Diagnostic Questionnaire for Internet Addiction score was 3.27 (SD = 1.81) and the mean PHQ-9 score was 7.00 (SD = 4.24). Overall, 24.4% and 28.8% of the students had possible IA and depression, respectively. Nearly three fourths of all participants spent around three hours per day or more on the Internet, and around one quarter spent more than five hours per day. Using the Internet longer than five hours a day was significantly associated with both possible IA (OR 2.98, 95%CI: 1.77–4.99, P-value<0.01) and depression (OR 2.48, 95%CI: 1.58–3.90, P-value<0.01). A significant association between possible IA and depression was also observed (OR 1.92, 95%CI: 1.34–2.77, P-value<0.001).

**Table 1:** Demographic characteristics and their associations with possible Internet addiction and depression.

		Possible Internet addiction N(%)	Normal Internet use N(%)	OR (95%CI)	Depression N(%)	No depression N(%)	OR (95%CI)
**Sex**	female	108 (27.8)	281 (72.2)	1.46 (1.05–2.13)^b^	117 (30.3)	269 (69.7)	1.16 (0.84–1.62)
	male	64 (20.4)	249 (79.6)		85 (27.2)	227 (72.8)	
**Academic level**	pre-clinical years (1^st^-3^rd^)	100 (24.6)	307 (75.4)	1.00 (0.71–1.42)	121 (29.7)	286 (70.3)	0.92 (0.66–1.28)
	clinical years (4^th^-5^th^)	111 (33.1)	224 (66.9)		82 (28.0)	211 (72.0)	
**Grade point average (GPA)**^c^	> = 3.5	51 (27.0)	138 (73.0)	0.99 (0.56–1.77)	47 (25.0)	141 (75.0)	0.57 (0.33–0.99)^b^
	3.00–3.49	55 (21.4)	202 (78.6)	0.73 (0.42–1.29)	67 (26.1)	190 (73.9)	0.60 (0.36–1.02)
	< = 2.99	23 (27.1)	62 (72.9)		31 (36.9)	53 (63.1)	
**Comorbid medical illness**	yes	39 (25.3)	115 (74.7)	1.05 (0.70–1.59)	51 (33.3)	102 (66.7)	1.30 (0.88–1.90)
	no	134 (24.4)	415 (75.6)		152 (27.8)	394 (72.2)	
**Alcohol drinking**^d^	> once per day	16 (23.0)	53 (76.8)	0.87 (0.48–1.58)	19 (29.2)	46 (70.8)	1.03 (0.58–1.82)
	< once per day	33 (21.7)	119 (78.3)	0.80 (0.52–1.24)	50 (34.7)	94 (65.3)	1.32 (0.89–1.97)
	no drinking	124 (25.7)	358 (74.3)		134 (28.7)	333 (71.3)	
**Average time of Internet use per day**^e^	> 5 hours	60 (33.1)	121 (66.9)	2.98 (1.77–4.99)^a^	77 (43.3)	101 (56.7)	2.48 (1.58–3.90)^a^
	3–4 hours	85 (25.1)	253 (74.9)	2.02 (1.24–3.27)^b^	81 (24.1)	255 (75.9)	1.03 (0.68–1.58)
	< 2 hours	26 (14.3)	156 (85.7)		43 (23.5)	140 (76.5)	

^a^ P-value < 0.01

^b^ P-value < 0.05

^c^ OR compared to “< = 2.99” group

^**d**^ OR compared to “no drinking” group

^**e**^ OR compared to “< 2 hours” group

Table 2 demonstrated participants’ response to the Young Diagnostic Questionnaire for Internet Addiction and associations between each item of the questionnaire and depression. Over 60% of the participating students in both depression and no depression groups reported that they used the Internet to avoid facing problems or to ameliorate negative emotions. More than 80% in both groups reported surfing online longer than they had intended. Almost all items showed statistically significant associations between positive results or “yes” responses on all individual items and depression, except item 8. The strongest association was found for item 6 (OR 2.16, 95%CI: 1.46–3.19, P-value<0.001).

**Table 2:** Associations between the Young Diagnostic Questionnaire for Internet Addiction items and depression.

Items	% “Yes” response Depression	% “Yes” response No depression	OR (95%CI)	P-value
**1. Do you feel preoccupied with the Internet (think about previous online activity or anticipate next online session)?**	59.6	49.4	1.51 (1.09–2.11)	0.014
**2. Do you feel the need to use the Internet with increasing amounts of time in order to achieve satisfaction?**	41.9	27.6	1.89 (1.34–2.66)	<0.001
**3. Have you repeatedly made unsuccessful efforts to control, cut back, or stop Internet use?**	52.7	43.3	1.46 (1.05–2.02)	0.024
**4. Do you feel restless, moody, depressed, or irritable when attempting to cut down or stop Internet use?**	25.6	15.3	1.90 (1.28–2.84)	0.001
**5. Do you stay online longer than originally intended?**	87.2	80.4	1.66 (1.04–2.64)	0.033
**6. Have you jeopardized or risked the loss of a significant relationship, job, educational, or career opportunity because of the Internet?**	28.1	15.3	2.16 (1.46–3.19)	<0.001
**7. Have you lied to family members, a therapist, or others to conceal the extent of involvement with the Internet?**	17.7	10.9	1.76 (1.12–2.79)	0.014
**8. Do you use the Internet as a way of escaping from problems or of relieving a dysphoric mood (e.g., feelings of helplessness, guilt, anxiety, depression)?**	70.0	62.3	1.41 (0.99–2.00)	0.055

When stress-related factors were analyzed, academic and health problems were the only two issues that showed significant associations with possible IA (OR 2.12, 95%CI: 1.40–3.20, P-value<0.01; and OR 1.72, 95%CI: 1.07–2.76, P-value<0.05, respectively). Difficulties in various aspects of life were all associated with depression, but conflicts with teachers appeared to be the most significant one (OR 8.60, 95%CI: 2.34–31.60, P-value<0.01) (Table 3).

**Table 3:** Factors associated with stress in medical students and their associations with possible Internet addiction and depression.

	Possible Internet addiction N(%)	Normal Internet use N(%)	OR (95%CI)	Depression N(%)	No depression N(%)	OR (95%CI)
**Academic problems**	47 (36.7)	81 (63.3)	2.12 (1.40–3.20)^a^	73 (57.5)	54 (42.5)	4.61 (3.08–6.90)^a^
**Difficulty in peer relationships**	17 (28.3)	43 (71.7)	1.24 (0.69–2.24)	38 (63.3)	22 (36.7)	5.02 (2.89–8.74)^a^
**Difficulty in love relationships**	13 (29.5)	31 (70.5)	1.31 (0.67–2.57)	25 (56.8)	19 (43.2)	3.59 (1.93–6.68)^a^
**Health problems**	31 (34.1)	60 (65.9)	1.72 (1.07–2.76)^b^	53 (58.2)	38 (41.8)	4.32 (2.74–6.81)^a^
**Financial problems**	12 (26.7)	33 (73.3)	1.13 (0.57–2.25)	21 (47.7)	23 (52.3)	2.39 (1.29–4.43)^b^
**Family problems**	8 (21.6)	29 (78.4)	0.84 (0.38–1.88)	23 (62.2)	14 (37.8)	4.45 (2.24–8.84)^a^
**Conflict with teachers**	5 (38.5)	8 (61.5)	1.95 (0.63–6.04)	10 (76.9)	3 (23.1)	8.60 (2.34–31.60)^a^

^a^ P-value < 0.01

^b^ P-value < 0.05

### Predictors of possible IA and depression

To study what factors could be predictors for possible IA and depression, logistic regression analysis was used. The results revealed that medical students with possible IA had a 1.58-time chance to have depression compared to those without the condition (95%CI: 1.04–2.38, P-value = 0.031). In contrast, depression was not found to be a predictor of possible IA. Considering average time of Internet use, the students spending three to four hours per day on the Internet had a 2.01-time probability to have possible IA as compared to those spending less than two hours per day. When Internet consumes greater than five hours a day, the probabilities of having possible IA and depression were respectively 2.64 and 2.20 folds of those using less than two hours a day (Tables 4 and 5).

**Table 4:** Predictors for possible Internet addiction analyzed by logistic regression.

		Adjusted OR	95%CI	P-value
**Depression**		1.44	0.96–2.16	0.081
**Sex**		1.50	1.04–2.17	0.030
**Average time of Internet use per day**^a^	> 5 hours	2.64	1.55–4.49	<0.001
	3–4 hours	2.01	1.23–3.28	0.005
	< 2 hours			
**Academic problems**		1.79	1.12–2.87	0.015
**Health problems**		1.13	0.67–1.93	0.661

^a^Adjusted OR compared to “< 2 hours” group

**Table 5:** Predictors for depression analyzed by logistic regression.

		Adjusted OR	95%CI	P-value
**Possible Internet addiction**		1.58	1.04–2.38	0.031
**Average time of Internet use per day**^a^	> 5 hours	2.20	1.33–3.62	0.002
	3–4 hours	0.94	0.59–1.50	0.798
	< 2 hours			
**Academic problems**		2.67	1.68–4.26	<0.001
**Difficulty in peer relationships**		2.28	1.17–4.42	0.015
**Difficulty in love relationships**		2.29	1.12–4.68	0.023
**Health problems**		2.22	1.30–3.79	0.004
**Financial problems**		1.05	0.50–2.19	0.905
**Family problems**		1.74	0.74–4.07	0.204
**Conflict with teachers**		1.75	0.38–8.12	0.473

**^a^**Adjusted OR compared to “< 2 hours” group

Further analysis on factors associated with stress in medical students showed that academic problems were a significant predictor of both possible IA (OR 1.79, 95%CI: 1.12–2.87, P = 0.015) and depression (OR 2.67, 95%CI: 1.68–4.26, P<0.001). Additional predictors for depression also included difficulty in peer relationships (OR 2.28, 95%CI: 1.17–4.42, P = 0.015), difficulty in love relationships (OR 2.29, 95%CI: 1.12–4.68, P = 0.023), and health problems (OR 2.22, 95%CI: 1.30–3.79, P = 0.004).

## Discussion

This study aimed to explore the extent of possible IA as well as its association with depression among medical students in Thailand. Interestingly, the prevalence of possible IA in our study was found to be higher than those of IA observed by similar studies in Chile (11.5%) [14], Iran (10.8%) [15], and Greece (5.6%) [16]. In contrast, a study in Nepal reported almost twice the prevalence of possible IA in Thailand (44.6%) [13]. These discrepancies can be explained in part by the different of assessing instruments and case definitions used in each study. While the other studies used the Internet Addiction Test and classified only moderate to severe dependence as IA; ours used Young Diagnostic Questionnaire for Internet Addiction, which lacks severity grading, to specify IA. In addition, distinctive cultural context might be another important factor that could influence patterns of Internet use across different countries.

From our study, the odds of possible IA among those using the Internet longer than five hours a day appeared slightly greater than among those using three to four hours a day. Such finding may imply that the more time the students spending online, the more chance they become addicted. Moreover, spending more than five hours a day online also increased both likelihoods of having possible IA and depression, suggesting that this critical period might serve as a cutoff value for potentially problematic Internet use among Thai medical students.

Focusing on the relationship between possible IA and depression, this study observed a significant association between the two conditions, which is concordant with a number of prior studies [6–12]. Interestingly, we found that the pattern of using the Internet to cope with problems and emotional difficulties, as inquired in item 8 of the Young Diagnostic Questionnaire for Internet Addiction, was not associated with depression; which differed from all the other items. In particular, item 6, which asks about the history of negative consequences of Internet use on various life aspects and seems to be the most obvious evidence of problematic use, showed the strongest association with depression. From these observations, it may be implied that the use of the Internet as a coping strategy might not be pathological per se, but the pattern of uncontrollable, compulsive use could be linked to adverse outcomes like depression.

Despite a number of consistent reports for an association between IA and depression, mechanism underlying such relationship currently remain obscured. It has been proposed that IA may result in ineffective coping and difficulties in real life [23], which could then deteriorate daily life function, academic performance and relationships with others; finally, depression might develop. Alternative explanation is that IA and psychiatric symptoms may increase vulnerability to each other and/or share risk factors, leading to the onset or persistence of both conditions [24]. Indeed, genetic studies have revealed that a 5HTTLPR polymorphism that affects serotonin function was associated with depression as well as IA [25,26], suggesting that serotonin dysfunction could serve as a biological link between these two conditions. Nevertheless, further logistic regression analysis in this study revealed that possible IA could predict depression but not in reverse. From these data, it could conceivably be hypothesized that IA might be a precipitating factor for depression in medical students. Our hypothesis is supported by a prospective study showing that young people initially free of mental health problems but using the Internet pathologically could finally develop depression [27]. Moreover, analyses on stress-related factors also revealed that academic difficulties, which were previously reported as a leading cause of stress among medical students [19], exhibited the strongest association with depression and also predicted the probability of having possible IA. Taken these data together, we speculate that medical students may initially surf the Internet to relieve psychological stress from their study but then could turn into compulsive use, which negatively impacts even more on daily life functions and academic performance. As a result, such maladaptive coping ultimately lead to development of depression in return.

The most apparent strengths of this study were the large number of medical students being enrolled and a high rate of survey response, which might be partly due to our use of simple screening questionnaires that could shortly be completed. Therefore, the outcomes of the study could be representative of all the population. Moreover, to our knowledge, this is the first research to explore the association between IA and depression in Thai medical students. Our findings may trigger serious concerns about the significance of these prevalent problems in Thailand, which could lead to initiation of appropriate surveillance and interventions for this particular population.

### Study limitations

This study also has some limitations that should be noted. First, since the definition of IA has still been debatable, we could only define “possible IA” according to a cutoff criterion similar to the original study by Young [4] but could not certainly identify cases with “definite IA”. Secondly, as a cross-sectional observation, our work could not determine causal relationship between possible IA, depression and their associated factors. We suggest that a prospective study is still warranted to prove the hypotheses of causality as proposed in this study. Thirdly, we used self-reported questionnaires, which depend on respondents’ subjective self-introspection, so the response bias cannot be avoided. Furthermore, a dichotomous nature of response options about IA could lead to acquiescence bias and also limited detection of IA with different severity in our study. Lastly, other potential confounders involving inter-individual differences, like academic burden at the time of data collection, the nature of Internet activity, level of self-esteem, social skills and coping styles, for instance, were not included in our analyses. Thus, further studies are required.

## Conclusion

IA was likely to be a common psychiatric problem among Thai medical students. This study has shown that possible IA appeared to be associated with depression and academic difficulties, but further prospective studies are required to determine the causal relationship between IA and depression. We also suggest that surveillance of IA should be considered in medical schools.
